# The impact of dance activities on social skills and related behaviors in children and adolescents with autism spectrum disorders: a meta-analysis

**DOI:** 10.3389/fpsyt.2026.1805431

**Published:** 2026-05-19

**Authors:** Yi Jingyao, Song Dongpo, Liu Wanxu, Li Xiaofen

**Affiliations:** 1 School of Arts, Beijing Sport University, Beijing, China; 2 School of Physical Education, Changsha University of Science and Technology, Changsha, China

**Keywords:** autism spectrum disorder, children and adolescents, dance activities, meta-analysis, social skills

## Abstract

**Objective:**

This study aims to quantitatively assess the effects of dance activities on social skills and related behaviors in children and adolescents with autism spectrum disorder through a meta-analysis, thereby providing evidence-based support for clinical interventions.

**Methods:**

We searched eight Chinese and English databases—PubMed, Embase, Cochrane Library, EBSCO, Web of Science, China National Knowledge Infrastructure, Wanfang data, and VIP databases—up to March 20, 2025, for relevant literature and included randomized controlled trials that met the PICOS criteria. Two researchers independently conducted duplicate checks, double-blind screening, and data extraction using NoteExpress software. Quality was assessed using the Cochrane tool, and meta-analysis, subgroup analysis, sensitivity analysis, and publication bias testing were performed using RevMan 5.4 and Stata 18.0 software. The standardized mean difference (SMD) and 95% confidence interval (CI) were calculated to assess the effect size.

**Results:**

A total of 14 studies involving 312 participants were included. The meta-analysis showed that dance activities effectively improved social skill impairments in children and adolescents with autism [SMD = −1.96, 95% CI: −2.63 to −1.28, p < 0.00001], as well as communication skills [SMD = −1.87, 95% CI (−2.75, −0.99), p < 0.0001], social interaction [SMD = −2.04, 95% CI [−2.99, −1.09], p < 0.0001], repetitive behaviors [SMD = −1.50, 95% CI [−2.23, −0.77], p < 0.0001], and perceptual abilities [SMD = -1.64, 95% CI (-2.16, -1.12), p < 0.00001] showed positive improvements, but there was high heterogeneity among studies. Subgroup analysis suggests that age may influence the effectiveness of the intervention.

**Conclusion:**

Dance activities may positively impact social skills and related behaviors in children and adolescents with autism spectrum disorder, with younger individuals showing more pronounced benefits. Optimal outcomes can be achieved through strategies such as incorporating peer interactions, extending intervention duration, and integrating medication management. However, caution is warranted in interpreting these findings due to significant sample heterogeneity and insufficient sample sizes. Future research should design more high-quality randomized controlled trials to further validate these effects.

**Systematic review registration:**

https://www.crd.york.ac.uk/prospero/, identifier CRD420251015850.

## 1 Introduction

Autism spectrum disorder (ASD) is a neurodevelopmental disorder characterized primarily by impairments in social interaction and communication, as well as repetitive and stereotyped behaviors (1). In recent years, its global prevalence has significantly increased (2–4), imposing a heavy burden on families and society (5). While existing intervention methods can improve certain symptoms in individuals with autism to some extent, they remain limited in enhancing social skills. This has prompted researchers to continuously explore innovative adjunctive intervention methods to address the shortcomings of traditional therapies.

Dance activities are defined by academia as a multidimensional practice integrating cultural, expressive, and physiological dimensions. According to anthropological definitions, dance constitutes a purposeful, consciously rhythmic, and culturally patterned sequence of nonverbal bodily movements. The American Dance Therapy Association (ADTA) further elaborates from a psychological intervention perspective, emphasizing that Dance/Movement Therapy (DMT) involves the psychotherapeutic application of movement to promote integration across emotional, social, cognitive, and physical dimensions. Neural mechanism research (6) indicates that dance activates the mirror neuron system during movement observation and execution, thereby enhancing social cognition and emotional resonance capabilities. In summary, as a structured mind-body intervention, dance’s core components primarily include: rhythmic bodily movement, musicality, nonverbal expression, social interaction, creativity and improvisation, and mind-body unity. Its efficacy stems from the synergistic interaction of these multifaceted elements, rather than isolated physical activity.

Dance activities, as an emerging and creative therapeutic approach, integrate elements such as movement perception (7), musical rhythm, and emotional expression (8), providing individuals with ASD with a unique and comprehensive intervention platform (9). Through physical training, social interaction, and non-verbal communication, dance activities can create a structured, enjoyable, multi-sensory stimulating environment for individuals with ASD (10), which helps promote neuroplasticity, enhance social motivation, and improve social behavior. Researchers have found through scaffolding theory that dance activities can build dynamic learning scaffolds for children through progressive physical guidance and structured social support, gradually withdrawing assistance to achieve autonomous development of social skills (11). Preliminary exploratory studies indicate that dance activities have potential in enhancing the frequency of social interaction, accuracy of emotional recognition, and proactive communication among children and adolescents with ASD (12). Other studies suggest that dance, through synchronized movements, mirror neuron activation, and emotional empathy promotion, may help children with ASD overcome language barriers, enhance social attention, and improve cooperative abilities (13). Additionally, the embodied cognitive characteristics of dance activities emphasize the dynamic connection between bodily experience and cognitive development (14), providing theoretical support for reshaping the social information processing patterns of individuals with ASD. However, due to inconsistent research findings, the specific role and place of dance activities in ASD treatment remain undefined.

Art therapy has emerged as a hot research area in ASD intervention due to its non-invasive and expressive characteristics (15–17). As a multimodal intervention integrating physical movement, musical rhythm, and social interaction, dance intervention demonstrates unique potential. Some randomized controlled trials indicate that structured dance courses can significantly enhance emotional recognition and peer interaction skills in children with ASD (18), but their effects may be moderated by intervention duration, participant age, and severity of core symptoms. This heterogeneity stems from differences in research design and the diversity of dance intervention forms themselves.

In recent years, multiple systematic review studies have examined the effects of dance activities on the social skills of individuals with autism spectrum disorder. Building upon this research, the present study not only validated the overall positive effects of dance activities on social skills and related behaviors in individuals with autism but also revealed, through subgroup analyzes, how different intervention characteristics influence treatment outcomes. This provides valuable insights for optimizing multidisciplinary intervention designs and offers robust evidence-based support for personalized intervention strategies in clinical practice.

## 2 Methods

This meta-analysis, registered on PROSPERO (CRD420251015850), was conducted according to the updated Preferred Reporting Items for Systematic Reviews and Meta-Analysis statement.

### 2.1 Search strategy

This study conducted a systematic search of PubMed, Embase, Web of Science, Cochrane Library, China National Knowledge Infrastructure (CNKI), Wanfang, and VIP databases, with the search period spanning from the establishment of the databases to March 20, 2025. The search strategy was constructed using a combination of free-text terms and subject headings, with Boolean operators (AND/OR) used for logical operations. References from included studies were manually traced. The search was restricted to Chinese and English language publications, excluding studies in other languages (See **Supplementary Note** online).

### 2.2 Inclusion and exclusion criteria

Literature screening strictly follows the PICOS principle (see **Table 1** for details), with the following specific criteria (19):

**Table 1:** Inclusion and exclusion criteria for literature.

Principle	Inclusion criteria	Exclusion criteria
P (Population)	Children and adolescents (aged <18 years) with ASD diagnosed according to DSM-5* or clinical diagnosis	Combined with serious physical disability and age over 18
I(Intervention)	Intervention forms include dance therapy, creative dance, standardized dance training, etc.	Non-dance interventions and interventions that do not describe specific dance forms or intervention processes
C(Comparison)	Standard care, regular physical activity, or normal daily activities, etc.	Studies without a non-autistic group
O(Outcome)	Social skills scales (SRS* etc.) and observational social behavior indicators	Studies that did not report social skills-related outcomes Only qualitative descriptions or non-standardized assessment tools
S (Study design)	randomized controlled trial	Reviews, case reports, theoretical discussions, Studies without any control group (e.g. single-arm studies without a standard care group, a group engaging in routine physical activity, or a placebo control group).

*DSM-5 refers to the Diagnostic and Statistical Manual of Mental Disorders, Fifth Edition, which is the authoritative standard for diagnosing autism spectrum disorders published by the American Psychiatric Association.

*The Social Reaction Scale (SRS) is a measurement tool used to assess social skills and social functioning deficits in individuals with autism spectrum disorders. Due to the scarcity of dance intervention studies targeting the autism population, no specific outcome measures were set during the literature search phase to maximize inclusion of potential studies. However, through manual screening, studies lacking data on social skills and related behaviors were ultimately excluded to ensure the analysis remained focused on the core research question.

The inclusion criteria for this study were as follows: study participants were children and adolescents under the age of 18 diagnosed with ASD according to DSM-5 (20) or clinical diagnosis; intervention measures included dance therapy, creative dance, standardized dance training, etc.; control measures included standard care, routine physical activity, or a blank control; outcome measures used social skills scales (such as SRS, ASSS, etc.) and observational social behavior indicators; the study design was a randomized controlled trial.

Exclusion criteria include: participants with severe physical illnesses or aged over 18 years; studies involving non-dance interventions or those without clearly defined dance forms or intervention protocols; studies without non-autistic group; studies that did not report social skills-related outcomes or relied solely on qualitative descriptions or non-standardized assessment tools; reviews, case reports, theoretical discussions, or pre-post studies without non-autistic group.

### 2.3 Literature screening and data extraction

The literature screening and data extraction process for this study is as follows: The retrieved literature was imported into NoteExpress literature management software, and duplicate entries were deleted after automatic duplication checking. Two researchers (JY and DS) independently conducted a double-blind screening process: in the first stage, potential eligible literature was preliminarily screened based on titles and abstracts; in the second stage, the full text was read to confirm eligibility for inclusion. Disagreements were resolved by a third researcher (XL) through negotiation or arbitration: XL first facilitated discussions between the two reviewers to seek consensus. If consensus could not be reached, XL acted as the arbitrator to make the final decision. Data extraction was conducted using standardized forms covering author, publication year, sample size, intervention protocol (duration/frequency/format), relevant outcome measures, and methodological quality elements. The extraction process also employed a double-checking and cross-validation mechanism to ensure data completeness and accuracy.

### 2.4 Study quality assessment

This study employed the Cochrane Risk of Bias Assessment Tool to evaluate the methodological quality of included randomized controlled trials (21). The Cochrane RoB 1.0 tool assesses seven domains: random sequence generation, allocation concealment, blinding of participants and personnel, blinding of outcome assessment, completeness of outcome data, selective reporting, and other potential sources of bias. Given the inherent characteristics of exercise interventions (where double-blinding of both participants and personnel is unfeasible), the blinding of participants and personnel dimension was excluded from assessment. Each dimension was categorized as “low risk,” “high risk,” or “uncertain risk.” The assessment workflow proceeded as follows: First, two researchers (JY and DS) independently assessed each study’s six bias risk dimensions using standardized evaluation forms. Subsequently, their results were cross-checked and compared. In cases of disagreement, the two researchers first attempted to reach consensus through discussion. If consensus could not be reached, a third researcher (XL) was consulted for arbitration and final decision-making to ensure the objectivity of the assessment process and the reliability of the conclusions.

### 2.5 Statistical methods

Statistical analyzes for this study were performed using RevMan 5.4 and Stata 18.0 software. Effect sizes were calculated using standardized mean differences (SMDs) for continuous variables, with SMD values of 0.2–0.49, 0.5–0.8 and >0.8 considered to represent small, medium and large effects, respectively (22), and presented with 95% confidence intervals (95% CI). To account for potential small-sample bias, Hedges’ g correction was applied in the sensitivity analysis. In addition to reporting the I² statistic and the Q test, the heterogeneity assessment also reports τ² (between-study variance), which reflects the degree of variation in the true effect size across different studies. (I² ≥50% or P<0.10 indicates significant heterogeneity) (23). As the Q-test has low statistical power and is prone to failing to detect genuine heterogeneity, the threshold is relaxed to 0.10 in order to improve the test’s sensitivity to heterogeneity. Given the anticipated clinical and methodological differences among the included studies regarding the type of dance intervention, participants’ age, intervention dose, outcome measurement tools, and cultural background, this study prioritized the use of a random-effects model in all primary meta-analyzes, as this approach provides more conservative and generalizable effect size estimates in the presence of heterogeneity.

With regard to sources of heterogeneity, predefined subgroup analyzes were conducted to explore potential confounding factors. Sensitivity analyzes were performed using the ‘leave-one-out’ method to test the robustness of the results. All subgroup analyzes were pre-specified in accordance with the PROSPERO registration protocol. Four subgroup analyzes were conducted in this study to examine the effects of age, intervention dose, peer involvement and medication use on the efficacy of the intervention. To control for the risk of Type I errors arising from multiple comparisons, we applied the Bonferroni correction. Furthermore, to assess whether differences in effect sizes between subgroups were statistically significant, we performed tests for interaction between subgroups and reported the p-values for these interaction effects. Publication bias was quantitatively assessed through visual inspection of funnel plots and Egger’s linear regression test (24). If raw data from a study are missing, we will attempt to contact the authors to obtain them; if this proves unsuccessful, the study will be excluded from any specific analyzes requiring those data, and only the available data will be reported. The significance threshold was set at a two-sided P < 0.05, and all analyzes were conducted in accordance with the PRISMA guidelines and the Cochrane Handbook.

## 3 Results

### 3.1 Results of the literature search

**Figure 1:**
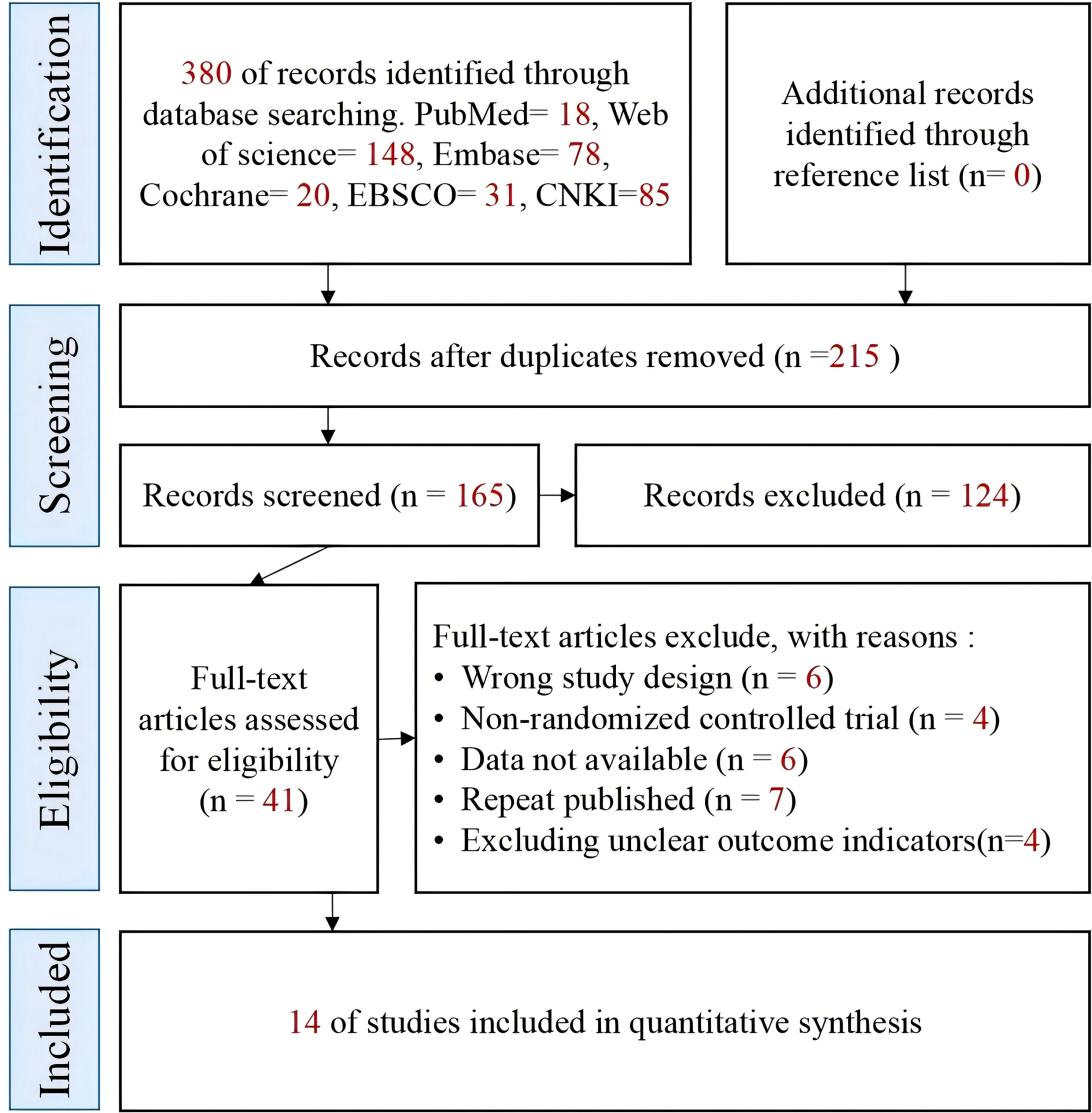
PRISMA flow diagram for the inclusion and selection of research.

This study conducted a systematic search of PubMed, Embase, Web of Science, Cochrane Library, China National Knowledge Infrastructure (CNKI), Wanfang, and VIP databases, yielding a total of 380 relevant literature sources. After automatic duplicate detection using NoteExpress software, 215 duplicate articles were excluded, leaving 165 articles for initial screening. Two researchers independently assessed the titles and abstracts, excluding 125 articles that were clearly irrelevant, conference abstracts, or non-English/Chinese language articles, retaining 41 articles for full-text review. Through full-text re-screening, four non-randomized controlled trials, six studies with incomplete data or unavailable full texts, seven duplicate publications, and ten studies with confounding intervention measures or inadequate study designs were further excluded. Ultimately, 14 randomized controlled trials (RCTs) meeting the PICOS criteria were included for analysis. The literature screening process is detailed in **Figure 1**.

### 3.2 Study characteristics

This study included a total of 14 studies (the main characteristics of the studies are summarized in **Table 2**), published between 2018 and 2024. The studies were geographically diverse, with one study each from the United Kingdom (29), Greece (38), Australia (30), and Iran (27), two studies from Brazil (33, 34), and eight studies from China (25, 26, 28, 31, 32, 35–37). These studies involved a total of 312 participants, with sample sizes ranging from 8 to 36 participants. The participants’ ages ranged from 5 to 18 years, and the intervention duration varied from 5 to 24 weeks. In terms of intervention measures, the experimental groups primarily focused on dance activities, including dance therapy, Greek traditional dance programs, dance-music combined therapy, children’s dance training, the ALLPLAY dance program, rumba, and aerobic dance; while the non-autistic groups received interventions such as physical exercise, standard care, or a waiting list control. In terms of outcome measures, four studies used the SRS scale, two used the ASSS scale, and the remaining studies employed tools such as the SCQ, TEACCH, and GARS-2. For the outcome measures, 9 studies focused on communication skills, 8 on interaction skills, 7 on repetitive behaviors, 6 on perceptual skills, and the remaining studies reported positive effects of dance activities on mood, cognition, and other aspects of children and adolescents with autism.

**Table 2:** Basic characteristics of literature.

Author, country, year	Age	Number of people(E/C)	Diagnostic methods	Methods of intervention(E/C)	Symptom severity	Medication use (yes/no)	Total length of intervention	Intervention frequency	Duration of intervention	Total intervention dose	Measure (p-value)	Peer involvement (yes/no)	Role
25	6-12	24(12/12)	Diagnosis certificate issued by the children’s hospital	DMT/SC	Mild/Moderate	No	12 weeks	3 times a week	80min	48h	SRS (p<0.01)	No	Parents &Teacher
26	5-8	14(7/7)	Diagnosed and confirmed by the Children’s Hospital	CDT/SC	Mild/Moderate	No	15 weeks	5 times a week	45min	56.2h	SRS (p<0.01)	No	Teacher
27	6-10	16(8/8)	Diagnosed by hospitals and trained researchers	CPT/SC	Moderate	No	8 weeks	1 times a week	60-80min	8-10.6h	GARS-2 (p<0.05)	No	Parents &Teacher
28	6-12	24(12/12)	Hold a certificate of autism diagnosis issued by a children’s hospital	DMT/SC	Mild/Moderate	No	12 weeks	3 times a week	80min	48h	SRS (p<0.01)	Yes	Parents &Teacher
29	8-13	26(10/16)	DSM-5	DMT/SC	Mild/Moderate /Severe	No	5 weeks	2 times a week	40min	6.6h	SCQ (p<0.05)	No	Parents
30	7-12	20(8/12)	Diagnosis certificate issued by the children’s hospital	AP/PE	Mild/Moderate	Yes	10 weeks	1 times a week	60min	10h	SRS-2 (p<0.05)	Yes	Parents
31	6-12	20(10/10)	Diagnosis certificate issued by the children’s hospital	DMT/CDT	Mild/Moderate	No	12 weeks	2 times a week	80min	32h	ASSS (p<0.05)	No	Parents &Teacher
29	6-14	8(4/4)	Diagnosis certificate issued by the children’s hospital	GTD/PE	Mild/Moderate	No	8 weeks	2 times a week	40min	10.6h	TEACCH (p<0.05)	No	Teacher
32	5-8	30(15/15)	DSM-5	DMT, MT/NI	Mild/Moderate /Severe	No	12 weeks	1 times a week	40min	8h	ATEC (p<0.05)	Yes	Parents
33	8-10	30(15/15)	Diagnosed by a psychiatrist	Dance/EAT	Mild	Yes	12 weeks	2 times a week	60min	24h	WHODAS 2.0 (p<0.05)	No	Teacher
34	8-15	36(17/19)	Diagnosed by a psychiatrist	Dance/SC	Mild/Moderate	Yes	24 weeks	1 times a week	40min	16h	ABC (p<0.01)	Yes	Parents
35	7-8	14(7/7)	Children Autism Rating Scale, CARS	CD/PE	Mild/Moderate	No	12 weeks	2 times a week	60min	24h	ASSS (p<0.05)	No	Teacher
36	6-12	20(10/10)	Diagnosis certificate issued by the children’s hospital	DMT/NI	Mild/Moderate	No	12 weeks	3 times a week	80min	48h	ATEC (p<0.01)	Yes	Parents
37	16-18	30(15/15)	Diagnosed by a psychiatrist	RD/PE	Mild/Moderate	No	12 weeks	3 times a week	40min	24h	BLERT (p<0.01)	Yes	Teacher

*DMT, Dance Movement Therapy; MT, Music Therapy; SC, Standard Care; CDT, Chinese Dance Training; CPT, Combined Physical Training; AP, ALLPLAY Dance; PE, Physical Exercise; GTD, Greek Traditional Dance; NI, No Intervention; EAT, Equine-Assisted Therapy; CD, Cheerleading Dance; RD, Rumba Dance.

### 3.3 Risk of bias

**Figure 2:**
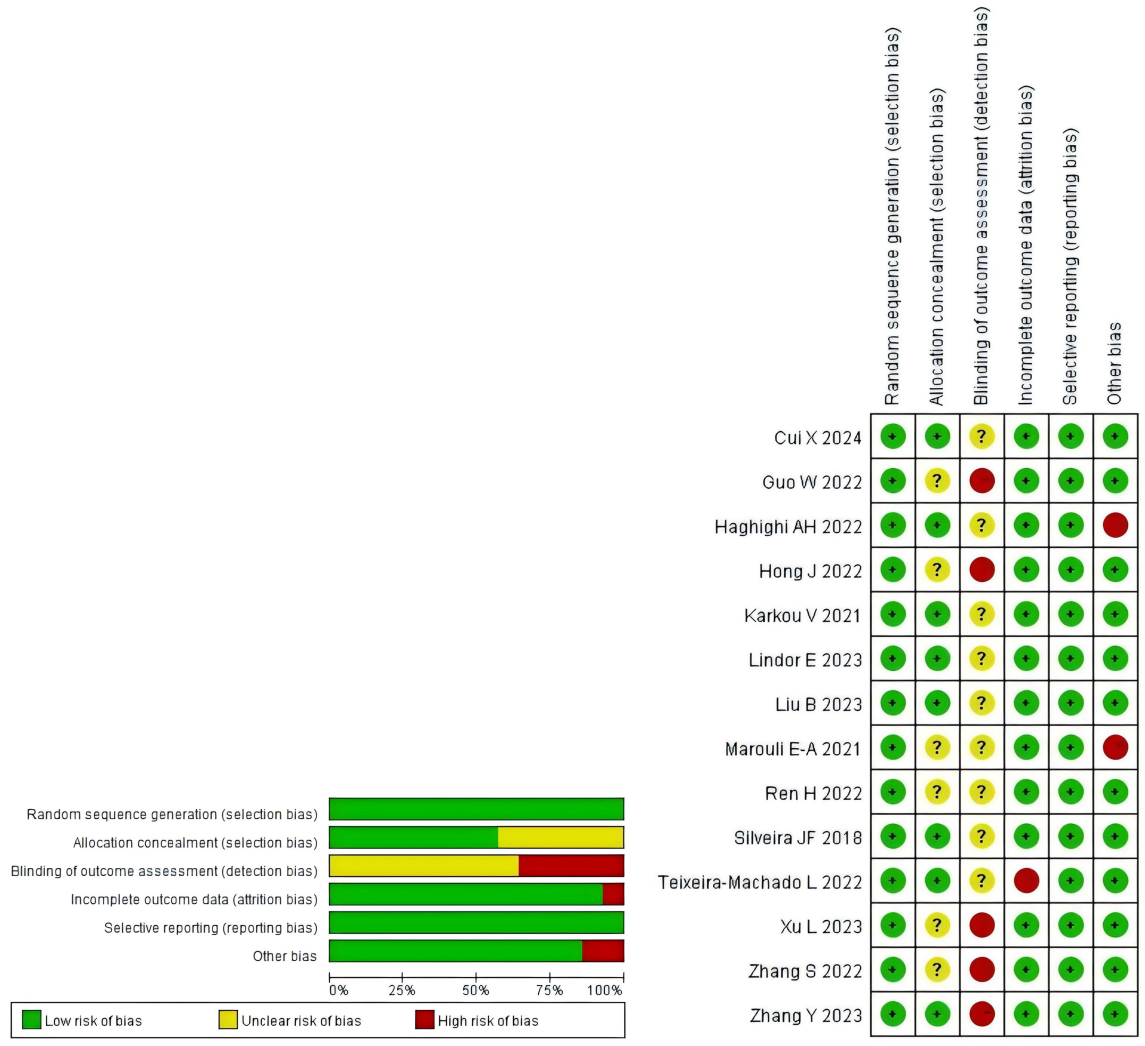
Risk of bias assessment results of included studies.

A methodological quality assessment of the 14 included studies was conducted using the Cochrane Risk of Bias Tool. All studies were rated as “low risk” for random sequence generation. Regarding allocation concealment, seven studies were judged as “low risk” based on explicit descriptions of appropriate methods (e.g., sequentially numbered, opaque, sealed envelopes), while the remaining seven were rated as “unclear risk” due to insufficient methodological details. The main risks of bias were concentrated in: (1) blinding of outcome assessment, where five studies were rated “high risk” due to lack of blinding and eight as “unclear risk” from inadequate reporting; (2) incomplete outcome data, with one study showing high attrition bias (>20% dropout) rated “high risk”; and (3) other biases, where two studies were deemed “high risk” due to very small sample sizes affecting baseline representativeness. Due to the inherent infeasibility of blinding participants and personnel in exercise interventions, performance bias was not assessed. Overall, the methodological quality of included studies was considered moderate. Summaries and visual representations of bias risk are presented in **Figure 2**.

### 3.4 Results of meta-analysis

This study included 14 randomized controlled trials designed to investigate the effects of dance activities on social skills and related behaviors in children and adolescents with autism. The results analysis included five independent meta-analyzes evaluating social skills and related behavioral measures, including language communication skills, social interaction, perceptual skills, and repetitive behavior scores. In addition to the Social Reaction Scale (SRS), other standardized scales are also used to assess social skills and related behavioral dimensions. Communication abilities were assessed using the Social Communication Questionnaire (SCQ). Social interaction was measured through observation and structured tools such as the TEACCH-based Social Interaction Scale. Repetitive behaviors were assessed using the Revised Repetitive Behaviors Scale (RBS-R) and relevant subscales from the Gilliam Autism Rating Scale-Second Edition (GARS-2). Perceptual abilities were evaluated through the Sensory Characteristics Questionnaire, aligned with a multisensory integration framework, and through operational tasks.

Before pooling data from different studies, it is essential to standardize data derived from various assessment tools to ensure consistency and comparability in effect size calculations, thereby eliminating computational biases stemming from differences in scale design. Specifically, this study employs the Standardized Mean Difference (SMD) as the effect size measure, calculated based on the mean, standard deviation, and sample size of each group. Due to variations in measurement direction across studies, we standardized the effect size calculations. For scales negatively correlated with social skills and related behaviors, we directly computed the Standardized Mean Difference (SMD). Conversely, for scales positively correlated with these outcomes, we calculated the SMD by reversing the order of group means. Therefore, in this study, lower scores indicate greater skill improvement.

#### 3.4.1 Total social skills score

**Figure 3:**
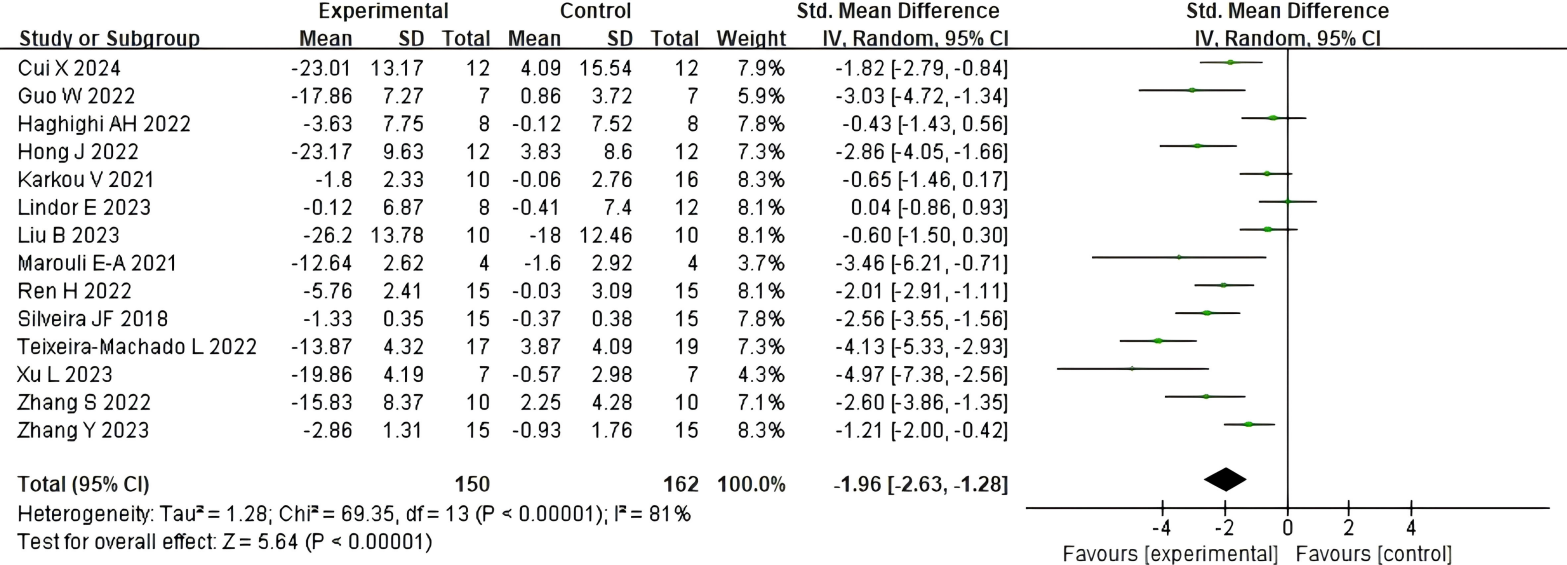
Impact of dance activities on overall social skills.

A total of 14 studies determined the effect of dance activities on total social skills scores through, among other things, the Social Responsiveness Scale (SRS), with scores negatively correlating with subjects’ social skills. The results showed a total social skills score effect size of [SMD = -1.96, 95% CI (-2.63, -1.28), p < 0.00001], with a high degree of heterogeneity and significance between the included studies (τ² = 1.43, I²= 81%, Chi2P < 0.00001), see **Figure 3**.

#### 3.4.2 Communication skills

A meta-analysis of 10 studies (n=220) revealed significant differences in communication skills between groups (SMD = -1.87, 95% CI [-2.75, -0.99], p < 0.0001), with high heterogeneity (τ² = 1.79, I² = 84%, Chi² p < 0.00001). Outcome measure scores were negatively correlated with communication skills, indicating that dance activities have a positive impact on communication skills in adolescents with autism.

#### 3.4.3 Social interaction

A meta-analysis of 8 studies (n=168) showed significant differences in interactive ability between groups (SMD = -2.04, 95% CI [-2.99, -1.09], p < 0.0001), with high heterogeneity (τ² = 1.59, I² = 80%, Chi² p < 0.00001). The outcome measures were negatively correlated with interactive ability, suggesting that the interactive abilities of adolescents with autism improved following dance activities.

#### 3.4.4 Repetitive behavior

A meta-analysis of 7 studies (n=128) showed that there were significant differences in repetitive behaviors between groups (SMD = -1.50, 95% CI [-2.23, -0.77], p < 0.0001), with high heterogeneity (τ² = 1.49, I² = 65%, Chi² p = 0.008). The outcome measures were negatively correlated with repetitive behaviors, indicating that dance activities improved repetitive behaviors in adolescents with autism.

#### 3.4.5 Perceptual abilities

A meta-analysis of 6 studies (n=122) revealed significant differences in perceptual abilities across groups (SMD = -1.64, 95% CI [-2.16, -1.12], p < 0.00001), with moderate homogeneity (τ² = 0.86, I² = 29%, Chi² p = 0.22). Outcome measure scores were negatively correlated with perceived ability, indicating that the social perceptual abilities of adolescents with autism can be enhanced through dance activities (See **Supplementary Figure 4** online).

### 3.5 Subgroup analysis

#### 3.5.1 The impact of dance activities on social skills in different age groups

**Figure 4:**
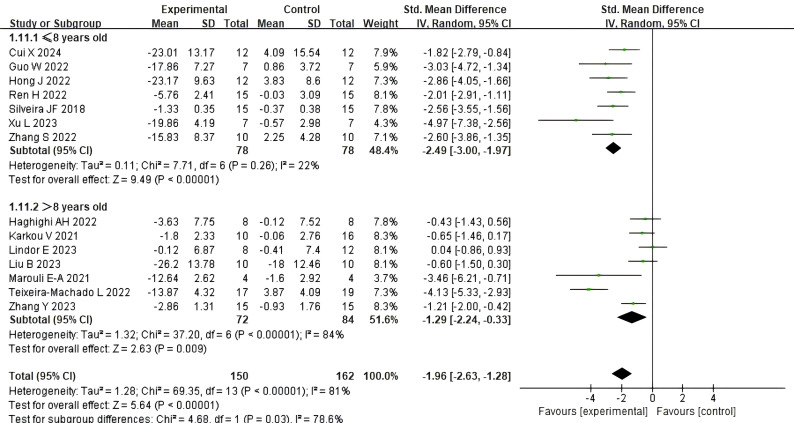
The impact of dance activities on social skills in different age groups.

**Figure 4** illustrate the impact of dance activities on the social skills of children and adolescents with autism across different age groups. A total of seven studies reported the intervention effects on participants aged 8 years and under, while seven studies reported the intervention effects on participants aged 8 years and above. Compared with the control group, dance activities were found to have a significant impact on the social skills of children and adolescents with autism aged 8 years and older (SMD = −1.29, 95% CI: −2.24 to −0.33, p = 0.009), with high heterogeneity (I² = 84%, Chi² p < 0.00001), and a larger and more significant effect was observed in the age group of 8 years and younger [SMD = −2.49, 95% CI (−3.00, −1.97), p < 0.00001], with moderate homogeneity (I² = 22%, Chi² p = 0.26). The analysis of variance revealed a statistically significant difference between the two groups (χ² = 4.68, df = 1, p for interaction = 0.03), suggesting that age is an important moderator of the intervention’s effectiveness.

#### 3.5.2 The impact of different intervention doses on social skills

**Figure 5:**
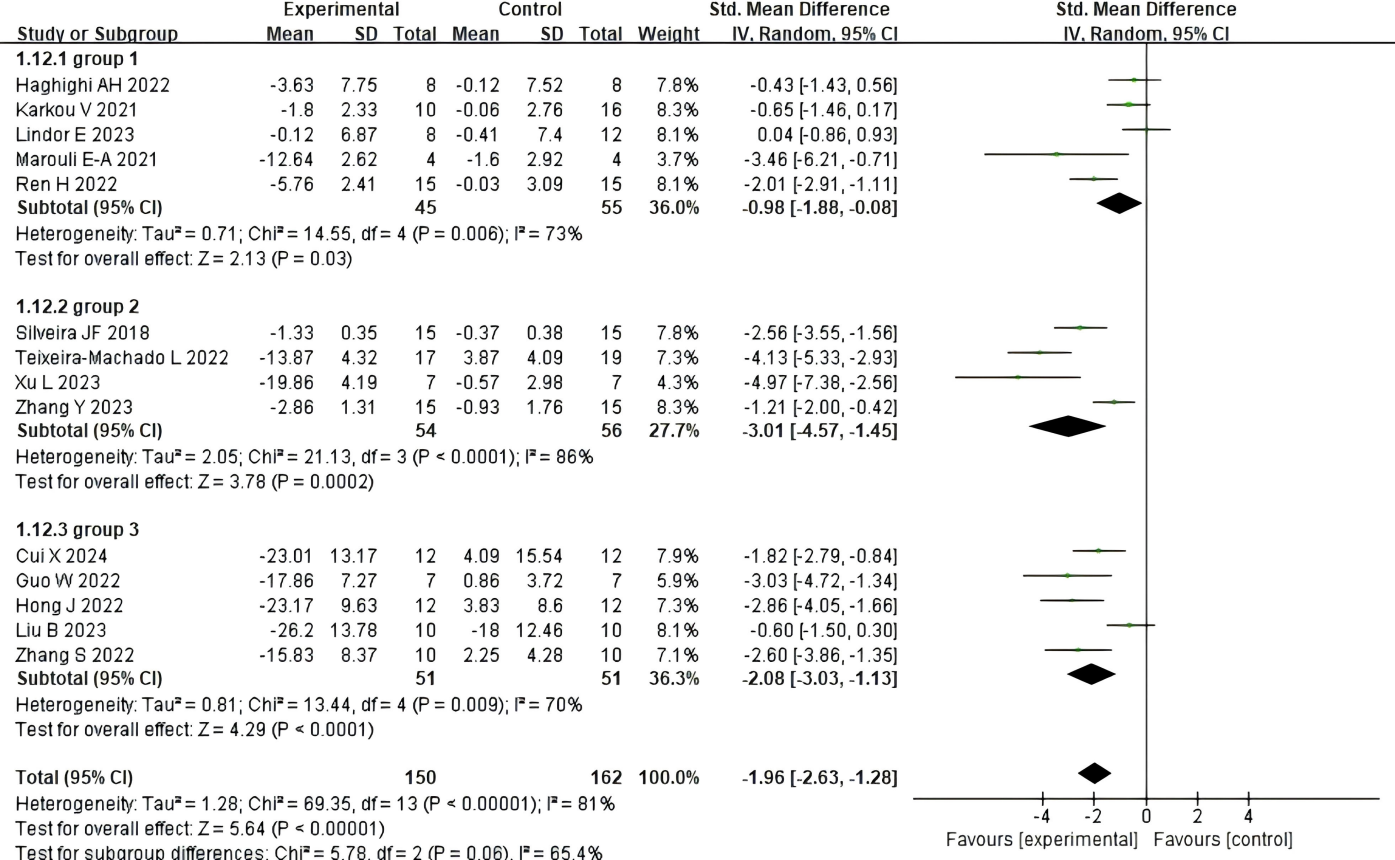
The impact of different intervention doses on social skills.

Following the Tertile Split method, we divided the total intervention dose (cumulative hours) into three groups: the low-dose group (Group 1, ≤10.6 hours), the medium-dose group (Group 2, 16–29.3 hours), and the high-dose group (Group 3, >29.36 hours). **Figure 5** illustrates the effects of different intervention doses of dance activities on social skills. Regarding intervention dose: the low-dose group intervention yielded an effect size of [SMD = −3.01, 95% CI (−1.88, −0.08), p = 0.03], with high heterogeneity (I² = 73%, Chi² p = 0.006); The medium-dose group intervention showed a more pronounced effect [SMD = −2.17, 95% CI (−4.57, −1.45), p < 0.00001], also exhibiting high heterogeneity (I² = 86%, Chi² p < 0.0001); The high-dose group intervention yielded an effect size of [SMD = −2.08, 95% CI (−3.03, −1.13), p < 0.0001], with high heterogeneity (I² = 70%, Chi² p = 0.009). The analysis of variance revealed that the differences in effect sizes across the three groups were not statistically significant (χ² = 5.78, df = 2, p for interaction = 0.06, I² = 65.4%), suggesting that, under the conditions of this study, the intervention dose did not exhibit a significant moderating effect.

#### 3.5.3 The impact of peer involvement during dance activities on social skills

**Figure 6:**
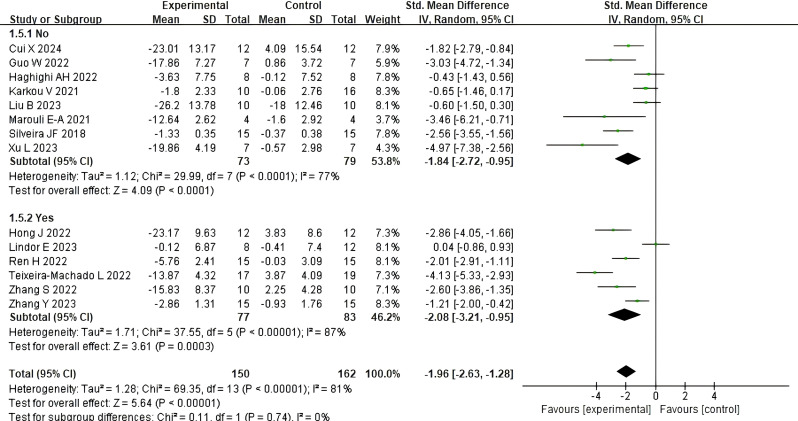
The impact of peer involvement during dance activities on social skills.

This study uniformly defines “peer” as an individual who is not the primary therapist or treatment provider, intentionally introduced into the intervention setting to interact with individuals on the autism spectrum (ASD). This category includes professionally trained volunteers and family members of participants. Due to variations across studies in describing peer backgrounds, training levels, and specific interaction methods, this study did not further differentiate peer characteristics in subgroup analyzes. Instead, peer involvement was recorded solely as “yes” or “no.” **Figure 6** illustrate the impact of peer involvement on social skills during dance activities. A total of 8 studies reported the effects of peer involvement during the intervention process, while 6 studies reported the effects of no peer involvement. The results showed that the absence of peer involvement during dance activities had a significant impact on social skills (SMD = −1.84, 95% CI: −2.72 to −0.95, p < 0.0001), with high heterogeneity (I² = 77%, Chi² p < 0.0001), while a larger and significant effect was observed in the presence of peer involvement (SMD = −2.08, 95% CI: −3.21 to −0.95, p = 0.0003), with high heterogeneity (I² = 87%, Chi² p < 0.00001). The analysis of variance revealed that no statistically significant difference in effect sizes between the two groups (χ² = 0.11, df = 1, p for interaction = 0.74, I² = 0%), suggesting that, under the conditions of this study, peer involvement did not exhibit a significant additional moderating effect.

#### 3.5.4 The impact of medication use during dance activities on social skills

**Figure 7:**
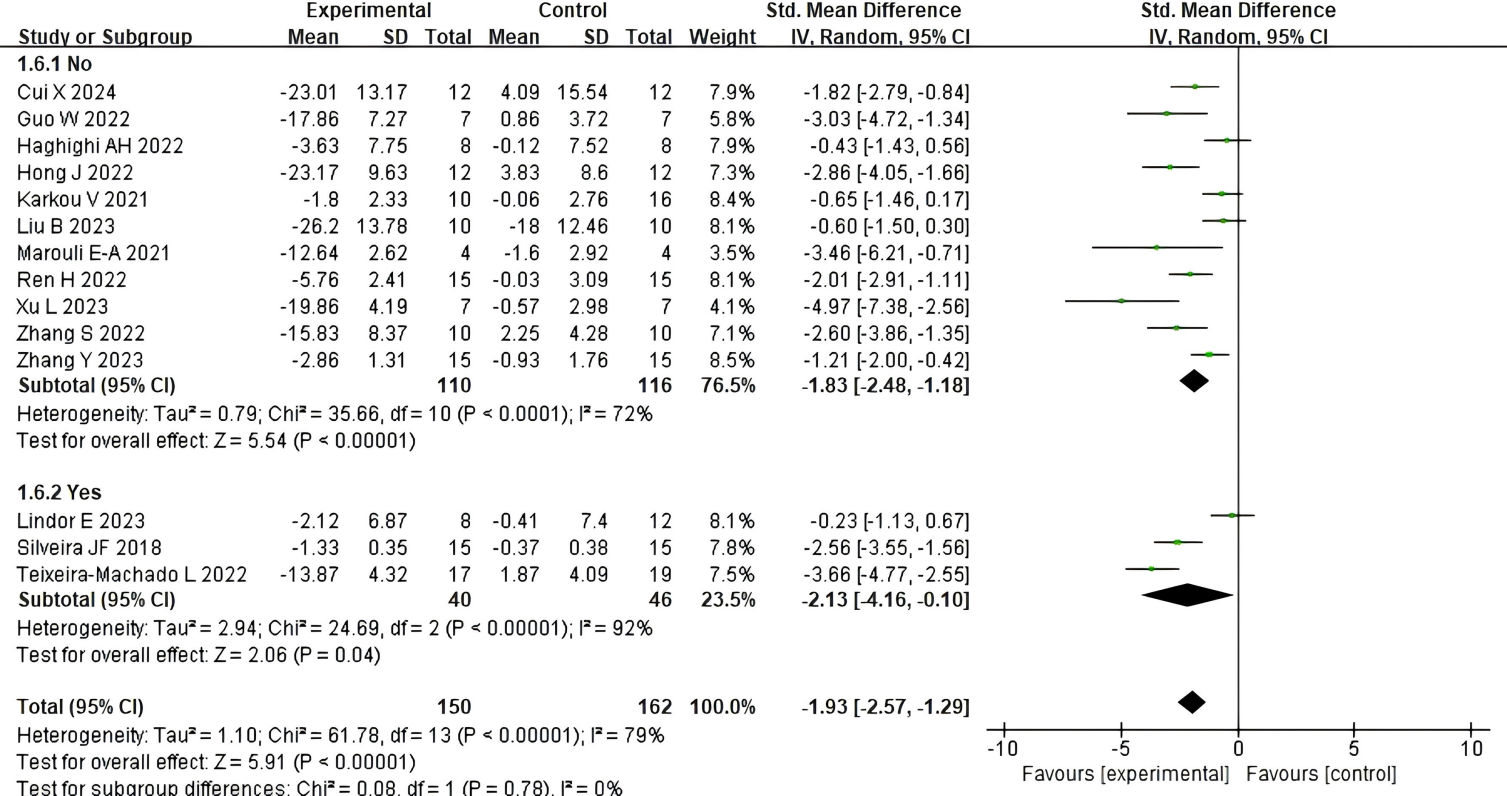
The impact of medication use during dance activities on social skills.

**Figure 7** illustrate the impact of medication use during dance activities on social skills. Four studies reported the effects of medication use during the intervention, while eight studies reported the effects of no medication use. The results indicated that no medication use during the intervention had a significant impact on social skills (SMD = −1.83, 95% CI: −2.48 to −1.18, p < 0.00001), with high heterogeneity (I² = 72%, Chi² p < 0.0001). In contrast, taking medication during the intervention had an even more significant effect (SMD = −2.13, 95% CI: −4.16 to −0.10, p = 0.04), with high heterogeneity (I² = 92%, Chi² p < 0.00001). The subgroup analysis revealed no statistically significant difference in effect sizes between the two groups (χ² = 0.08, df = 1, p for interaction = 0.78, I² = 0%), suggesting that, under the conditions of this study, medication use did not exhibit a significant additional moderating effect.

### 3.6 Meta-regression analysis

A meta-regression analysis examined whether variables such as publication year, intervention dose, mean age, sample size, medication use, and peer participation significantly moderated the overall effect size. Results indicated that none of these factors emerged as statistically significant moderators (p > 0.05).

### 3.7 Sensitivity analysis

**Figure 8:**
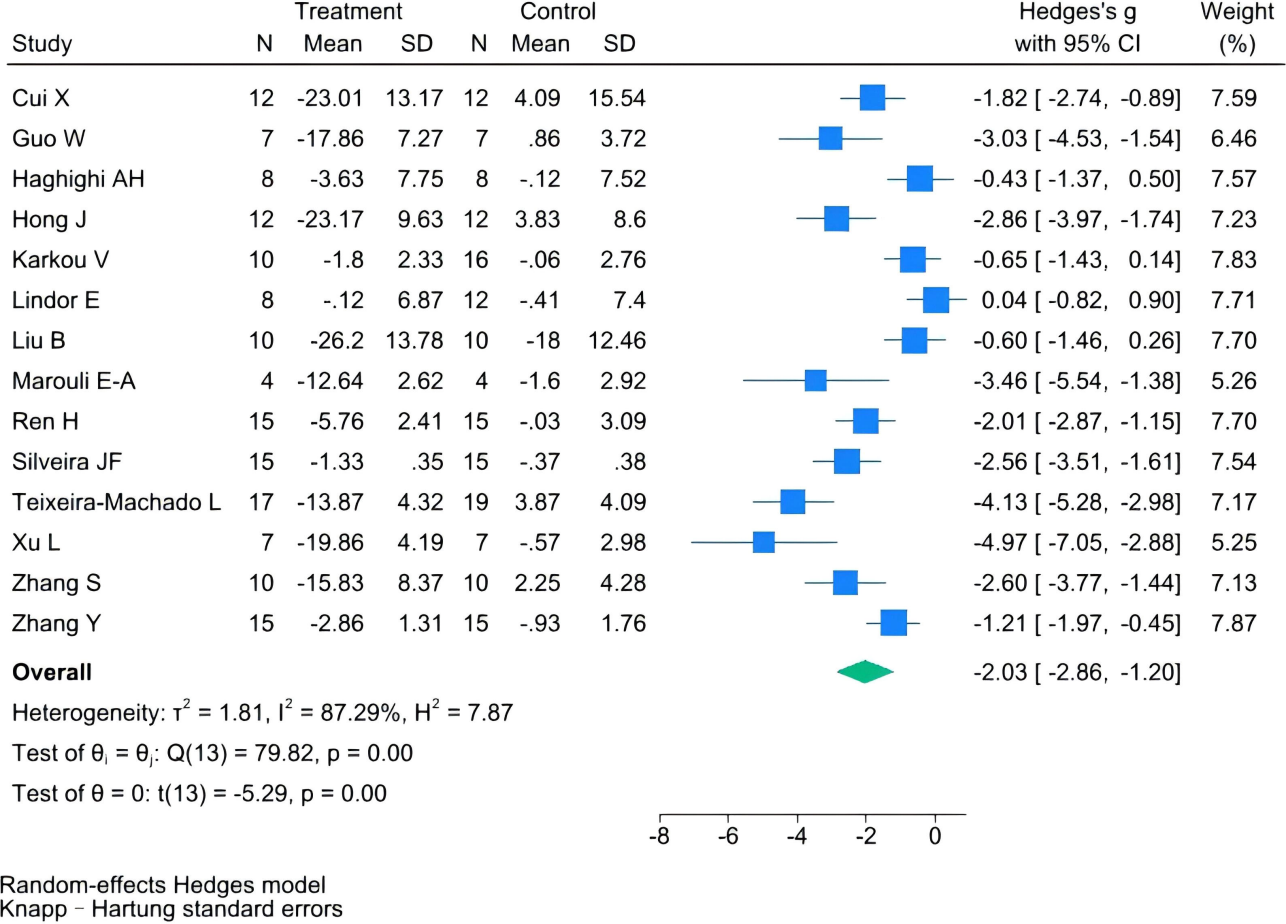
Meta-analysis results after applying the Hartung-Knapp correction.

**Figure 9:**
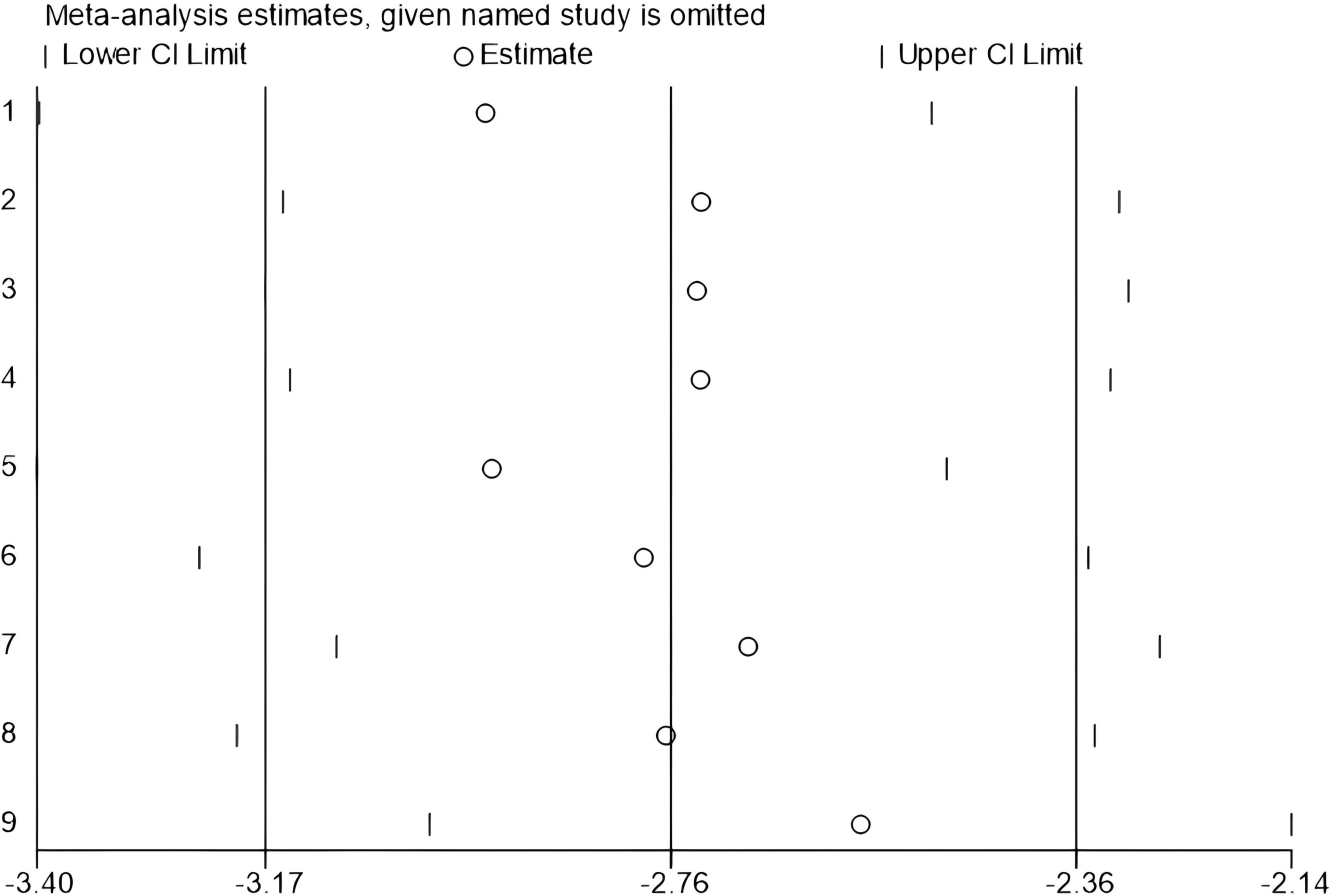
Impact on overall social skills after excluding 5 studies.

To assess the robustness of the pooled estimate and explore potential sources of heterogeneity, we conducted multiple sensitivity analyzes. First, results from the quality-weighted analysis based on Cochrane RoB 1.0 scoring indicated that studies with lower risk of bias demonstrated a more pronounced effect (SMD = –2.02, 95% CI: –2.36 to –1.68). Second, although Hartung-Knapp correction widened and made the confidence interval more conservative, it confirmed the stability of the results (SMD = –2.03, 95% CI: –2.86 to –1.20), We also recalculated all effect sizes using Hedges’ g to correct for small-sample bias. Finally, excluding five low-quality studies significantly reduced heterogeneity (I² = 46%, p = 0.06). The single-study exclusion analysis confirmed no single study exerted a disproportionate influence on the overall effect size, supporting the robustness of the primary findings (see **Figures 8**, **9**). Collectively, these sensitivity analyzes confirmed the stability of results despite existing heterogeneity.

### 3.8 Publication bias

This study further assessed the possibility of publication bias using funnel plots, Egger’s regression test, and trimming methods. The funnel plot showed mild asymmetry in the distribution of effect sizes for total social skills scores, and Egger’s test yielded a p-value of 0.032, suggesting the possibility of publication bias. After further adjustment using the trimming method, it was found that no missing studies needed to be imputed in the Stata random-effects model. The interpretation of the asymmetry in the funnel plot is inherently subjective, particularly in the presence of significant heterogeneity (I² = 81%). Given the clinical and methodological differences observed across the included studies, the asymmetry in the funnel plot is more likely to be attributed to heterogeneity rather than publication bias. This interpretation is supported by the ‘trim-and-fill’ method, which did not impute any missing studies. Consequently, the risk of publication bias in this study is low.

## 4 Discussion

This comprehensive meta-analysis included 14 empirical studies involving children and adolescents aged 5 to 18 years with autism spectrum disorder. Overall, dance activities not only have a positive impact on social skills [SMD = −1.96, 95% CI: −2.63 to −1.28, p < 0.00001] but also on communication skills [SMD = −1.87, 95% CI (−2.75, −0.99), p < 0.0001], social interaction [SMD = −2.04, 95% CI (−2.99, −1.09), p < 0.0001], repetitive behaviors [SMD = −1.50, 95% CI (−2.23, −0.77), p < 0.0001], and Perceptual Skills [SMD = -1.64, 95% CI (-2.16, -1.12), p < 0.00001] sub-dimensions all showed varying degrees of improvement effects. Our findings align with those of a recent systematic review (29) and meta-analysis (39, 40), further validating the effectiveness of dance activities in enhancing social skills among children and adolescents with autism.

Dance activities may have a promotional effect on the social skills of children and adolescents with autism spectrum disorder, with one proposed mechanism being the foundational role of rhythmic physical movement in development. Research suggests that rhythmic movement is associated with self-regulation and social communication skills (41). Dance activities, which often rely on imitation and synchronization, could enhance interpersonal motor coordination, potentially supporting emotional inference abilities in individuals with ASD. This offers a plausible basis for implicit interventions targeting social cognitive deficits (42). From a neurobiological perspective, it has been theorized—for instance, under social motivation theory—that social skill challenges in ASD may relate to differences in social motivation, which involve circuits such as the orbitofrontal cortex-striatum-amygdala network and related hormonal pathways (43). The amygdala is thought to play a central role in social information processing, including attentional orientation to social cues. Some studies propose that creative artistic activities (e.g., dance) may engage emotional regulation networks such as the medial prefrontal cortex-amygdala pathway (44), possibly enhancing emotional regulation and social interaction. As an embodied intervention, dance could facilitate bodily synchrony and activate social reward mechanisms, thereby compensating for motivational challenges in some individuals with ASD. Thus, dance activities integrate elements such as rhythmic synchrony, motor imitation, and potential neuroplasticity effects, offering a promising and practically valuable intervention avenue for ASD—particularly for those who may benefit less from traditional cognitive approaches (45). However, as none of the included studies directly assessed neural outcomes, the proposed neurobiological mechanisms remain speculative and warrant further empirical investigation.

However, the high heterogeneity among studies in this research indicates that clinical translation requires cautious consideration. To address this issue, we employed multiple analytical approaches: a quality-effect analysis based on Cochrane RoB 1.0 scoring, Hartung-Knapp correction to obtain more conservative confidence interval estimates, and meta-regression analysis to explore potential moderators. Results demonstrated consistent effect sizes across statistical methods, further confirming that despite heterogeneity, the intervention’s benefits may remain robust and reliable. Furthermore, while Egge tests and funnel plots indicated data asymmetry, the trim-and-fill method failed to identify studies requiring supplementation. This discrepancy likely stems from non-symmetrical data distributions caused by methodological bias (46). Schulz confirmed that trial design flaws systematically overestimate effect sizes and generate outliers with extreme heterogeneity. The resulting funnel plot asymmetry contradicts the trim-and-fill method’s assumption of “study symmetry” (47), potentially rendering the method ineffective at correcting bias. Although sensitivity analyzes indicate robust results, readers should exercise caution when interpreting them: the generally small sample sizes of the included studies may lead to a ‘small-sample effect’, thereby overestimating the true intervention effect of dance activities to some extent. Future large-sample, multicenter, and methodologically sounder randomized controlled trials are needed to validate the conclusions of this study and obtain more reliable estimates of effect sizes.

Similar to the findings of this study, (48) conducted a meta-analysis of 18 randomized controlled trials (RCTs) and demonstrated that music therapy (MT) yielded significant effects compared to control groups in improving language communication (SMD = -1.20), social skills (SMD = -1.13), behavioral performance (SMD = -1.92), sensory perception (SMD = -1.62), and self-care abilities (SMD = -2.14). However, the findings exhibited extremely high heterogeneity and a significant risk of publication bias. Persistently large effect sizes may reflect the genuine potential of arts interventions but could also stem from systematic bias. Therefore, while arts therapies hold promise for improving outcomes in autism spectrum disorders, these unusually high effect sizes warrant cautious interpretation—they may overestimate actual efficacy due to methodological limitations rather than therapeutic advantages.

Several factors may help explain the substantial heterogeneity observed in this meta-analytic results. For instance, The diversity of intervention formats represents a potential and significant source of heterogeneity in this study. The dance activities included in this study exhibit significant differences in their theoretical frameworks, core objectives and implementation methods. At one end of the spectrum lies dance/movement therapy (DMT), which is based on psychotherapeutic principles and aims to promote emotional regulation, social cognition and inner change through mind-body integration (49); at the other end are recreational dance forms that place greater emphasis on physical skills, coordination and fitness training, such as cheerleading, rumba and aerobic dance. This continuum ranging from clinical therapeutic interventions to physical activity-oriented interventions may result in different pathways of action and varying magnitudes of effect on social skills (50). Although this study analyzed all forms of dance activities as a single group, different types of dance interventions may trigger distinct therapeutic mechanisms, thereby contributing to the observed statistical heterogeneity. Future research should directly compare the differences in the effects of various dance forms on the social skills of children with autism spectrum disorder through pre-designed multi-arm comparative trials or network meta-analyzes.

Symptom severity may also contribute to variability in responses. Two studies included participants with severe symptoms (27) and (30), while others enrolled individuals with mild to moderate impairment. Some evidence suggests that milder autistic traits and younger age could be associated with greater intervention benefits, which appears consistent with earlier reports (51).

The presence of co-existing conditions, such as ADHD or Fragile X Syndrome, might further influence outcomes, potentially affecting attention, treatment adherence, or social functioning (52–55). The relationship between comorbidities and intervention efficacy is likely complex and may require individualized approaches (56). Future studies may benefit from more systematic reporting and consideration of co-existing conditions.

Variation in intervention dosage could represent another source of heterogeneity. Moderate-intensity, longer-term interventions may be especially suitable for improving social skills (57), whereas higher-intensity programs might support executive function but could also pose challenges for children with sensory sensitivities (58–60). Tailoring intensity based on individual profiles and response may help optimize benefits while reducing potential risks.

### 4.1 Communication skills

Communication difficulties are one of the core deficits of individuals with autism (61), and interventions utilizing rhythm have been shown to significantly enhance their communication abilities. This study also confirmed this finding. The results of this study indicate that dance activities have a positive impact on the communication abilities of children and adolescents with autism, with younger children showing more significant improvements in communication abilities, highlighting the importance of early intervention. Related studies have shown that participating in activities that involve synchrony, rhythm, and physical movement can effectively stimulate language abilities, motor skill development, and the activation of language-related brain networks in individuals with autism (62).

### 4.2 Social interaction

Dance activities synchronize musical rhythms with bodily movements, coordinating auditory, tactile, and kinesthetic sensory inputs to reduce hypersensitivity or under-responsiveness to environmental stimuli in individuals with autism. Such activities may activate the mirror neuron system, aiding in recognizing and responding to nonverbal social cues (63–65). Furthermore, rhythmic movements synchronized with music stimulate the release of neuropeptides such as oxytocin and vasopressin, thereby promoting social functioning (66). Collective dance experiences involving parents or teaching assistants can foster emotional resonance, enhancing social motivation. However, caution is warranted regarding potential responder bias when the implementer also serves as the evaluator. Future research should optimize intervention designs to minimize such biases, thereby more accurately assessing dance’s impact on social interaction.

### 4.3 Repetitive behavior

Repetitive behaviors, one of the core characteristics of individuals with autism, pose significant challenges to daily life and social adaptation. This study demonstrates that dance activities can effectively improve repetitive behaviors in children and adolescents with autism. The improvement of repetitive behaviors requires prolonged repetitive practice and habit restructuring. The rhythmic movement sequences in dance may indirectly reduce the frequency of repetitive behaviors by occupying children’s attention and physical activity time, a finding consistent with the results of (67). It is worth noting that dance designs targeting repetitive behaviors should avoid excessive structuring—overly mechanical movements may reinforce rigid patterns. Future recommendations suggest developing tiered intervention programs tailored to high-functioning and low-functioning individuals with autism: for low-functioning groups, focus on movement substitution training; for high-functioning groups, incorporate creative expression tasks to gradually expand behavioral flexibility. Through this individualized and multidimensional intervention strategy, dance activities hold promise as a comprehensive and effective treatment method for children and adolescents with autism, promoting the development of behavioral diversity and adaptive skills.

### 4.4 Perceptual abilities

Dance activities may offer benefits for perceptual challenges in autism. The Weak Central Coherence Theory suggests that individuals with autism may experience difficulties with multisensory integration, often focusing on local details rather than global information and showing challenges with audiovisual temporal processing, which might contribute to social withdrawal (68, 69). Dance combines rhythm, movement, and spatial awareness, potentially promoting neural coordination and supporting perceptual and attentional processes (70). Music-guided dance might also enhance neural synchrony and perceptual network efficiency (71), possibly improving adaptation to sensory environments. However, current evidence relies primarily on parent- or teacher-reported measures; incorporation of objective physiological indicators in future research could help validate these potential benefits.

### 4.5 Subgroup analysis

Subgroup analyzes aimed to investigate the effects of age, intervention dosage, peer participation, and medication use. Results revealed statistically significant differences across age groups (p=0.03), indicating that children under 8 years old demonstrated greater improvements in social skills compared to older participants. This finding aligns with (72), who noted that movement-based interventions yield more pronounced benefits for younger children. Early childhood may represent a window of heightened neuroplasticity, during which activities integrating sensory-motor and emotional elements—such as dance activities—can more effectively promote neural adaptation. Young children often demonstrate higher engagement in structured group settings, whereas adolescents with autism may exhibit reduced compliance due to heightened self-awareness. However, uneven sample distribution across age groups resulted in underrepresentation of older participants—a common limitation in ASD research (73). This asymmetry increases the risk of overestimating effects in younger children while failing to adequately capture potential benefits—or unique responsive patterns—in older age groups. Therefore, the apparent association between younger age and better outcomes may be partially confounded by imbalanced sampling and developmental differences in intervention engagement. Further studies with balanced age representations are needed to clarify the true effect of age on dance intervention efficacy.

Intervention dose was categorized into low, medium, and high tertiles based on cumulative hours. (74) found that higher doses of social skills interventions produced more sustained outcomes, while (10) reported that limited doses often failed to achieve clinical significance. Partially aligning with these findings, our analysis suggested that medium-dose interventions were associated with the largest effect sizes. Although this may highlight the importance of optimal dosing, substantial heterogeneity indicates that other factors beyond dose may influence outcomes. Therefore, medium dose should not be considered a universally optimal level. Future research should use individual participant data or prospective dose-response designs to identify personalized thresholds.

Peer-Mediated Intervention (PMI) appears to generate synergistic benefits. Compared to interventions without peer involvement, PMI enhances intervention effectiveness across all functional levels, confirming its role in promoting social interaction—as observed by (75), this approach proves particularly effective for individuals with lower functional levels. Therapist-guided peer synchrony training may help improve the inherent synchrony deficits in individuals with autism spectrum disorder (76), thereby promoting the development of empathy and social communication skills.

The effect size in the medication-assisted group was significantly higher than that in the medication-only group. This study found that combining dance activities with conventional medication treatment yields superior outcomes. This aligns with findings that integrating medication with behavioral training improves core symptoms of ADHD (77) and autism (78). However, it should be noted that the studies included in this analysis had a small sample size (k = 3), meaning these findings may not be broadly representative. Therefore, future research should further explore the optimal dosage and duration of medication interventions, investigate synergistic effects of medication combinations, and assess the impact of dosage adjustments on social skills.

## 5 Limitations

This study conducted a meta-analysis of 14 randomized controlled trials to comprehensively evaluate the effects of dance activities on social skills and related behaviors in children and adolescents with autism spectrum disorder. However, the study results have the following limitations: (1) this study only included studies written in Chinese or English, excluding conference papers, gray literature, unpublished studies, and studies written in other languages. This selection may introduce publication bias and language bias, resulting in the inability to cover all potential studies, thereby affecting the comprehensiveness and representativeness of the results; (2) the sample size of the included studies was small, and the proportion of the subgroup aged 8 years and above was relatively low, which may result in insufficient representativeness of the results. This age distribution imbalance may overestimate the intervention effects in the younger age group while neglecting the unique needs of adolescents and adults with ASD. Furthermore, small-sample studies often have lower methodological quality, which may further exaggerate the intervention effects.; (3) This study exhibits significant heterogeneity, and different assessment tools may introduce bias in efficacy evaluations. Although subgroup analyzes have explored certain variables, unidentified potential moderating factors may still exist. Furthermore, it must be acknowledged that randomized controlled trials based on dance activities inherently possess limitations, namely the inability to blind both participants and personnel. This limitation may lead to an overestimation of the intervention’s benefits; (4) In this study, some of the included randomized controlled trials exhibited certain methodological shortcomings, primarily in terms of the absence or lack of clarity regarding outcome assessment blinding, as well as inadequate allocation concealment. According to the Cochrane Risk of Bias Assessment Tool, these studies were rated as having a ‘high risk’ or ‘unclear risk’ for the two key items of ‘outcome assessment blinding’ and ‘allocation concealment’. Intervention studies of lower methodological quality tend to report larger effect sizes, which may have led to an overestimation of the overall effect size in this meta-analysis. Although heterogeneity decreased significantly and the overall direction of the effect remained unchanged after excluding five low-quality studies in our sensitivity analysis, the influence of methodological bias on the magnitude of the effect size cannot be entirely ruled out. Therefore, caution is warranted when interpreting the results of this study. Future research should include more high-quality randomized controlled trials with rigorous designs, blinded assessments and concealed allocation to further validate the true intervention effects of dance activities on the social skills of children and adolescents with autism spectrum disorder. (5) Another significant limitation of this study is the high level of heterogeneity among the included studies; the pooled effect size represents merely a statistical ‘average estimate’ and does not constitute a universal conclusion applicable to all forms of dance intervention. There may be significant differences in the impact of various dance activities on the social skills of children with ASD; standardized intervention protocols are required in future to reduce this heterogeneity. Furthermore, most of the original studies did not employ blinding of the assessors or provided unclear information regarding this; assessments of social skills relied heavily on the subjective judgements of parents or teachers. The assessors’ prior expectations may have introduced assessment bias, thereby overestimating the pooled effect size. Future research should strengthen the design of assessor blinding and supplement this with objective assessment methods such as standardized behavioral coding.

## 6 Implications for practice and research

The findings of this study indicate that dance activities can serve as a component of multidisciplinary intervention programs to improve social skills and related behaviors in children and adolescents with autism spectrum disorder. To translate this potential into actionable and scalable practice, the following implementation strategies are recommended: First, develop structured “dance prescriptions” for clinical settings, clearly defining intervention parameters—frequency, intensity (primarily moderate intensity while accommodating individual sensory sensitivities), and duration—to ensure effective doses for eliciting change. Second, the development of parent-child synchronized dance video tutorials for home use is encouraged. These resources should include clear visual demonstrations and rhythmic guidance to promote nonverbal interaction and enhance social synchrony in natural settings. Finally, a peer-mediated step-by-step training program can be developed for special education professionals. This program must define peer selection criteria, role assignments, and interaction strategies, incorporating scenario simulations and feedback mechanisms to ensure intervention fidelity. Despite these practical implications, interpreting the existing evidence requires particular caution. While the pooled effect size indicates a statistically significant effect, this magnitude is unusually large for psychosocial intervention studies and may reflect systematic bias in the included research rather than the true effect of dance activities themselves. Therefore, the core implication for future research is the urgent need for large-scale, rigorously designed randomized controlled trials. Such studies must employ blinded evaluators and effective control groups (e.g., standard social skills training) to control for non-specific effects of the intervention. This will yield more precise, unbiased, and clinically relevant effect size estimates, which are crucial for determining the precise role of dance activities within treatment protocols for autism spectrum disorders.

## 7 Conclusion

The findings of this study indicate that dance activities exert a positive influence on the social skills and related behaviors of children and adolescents with autism. Although current research is limited by small sample sizes and high heterogeneity, requiring cautious interpretation of conclusions, the results still suggest that dance activities hold promise as a valuable component within multidisciplinary intervention programs for autism. Future research should expand sample sizes and focus on establishing standardized intervention frameworks, while integrating clinical evidence from diverse linguistic and cultural contexts to enhance the applicability of findings. Furthermore, there is an urgent need to design high-quality randomized controlled trials that strictly control intervention dosage and ensure measurement tool consistency to systematically investigate the causal relationship between age and intervention outcomes. Subsequent research should also emphasize developing personalized treatment plans and conducting long-term follow-up studies. These efforts should systematically incorporate multilingual, multicenter collaborative data to advance dance activities from empirical application toward evidence-based, personalized, and multimodal intervention models.

## Data Availability

The original contributions presented in the study are included in the article/**Supplementary Material**. Further inquiries can be directed to the corresponding author.
