# Supplementary material for: The impact of dance activities on social skills and related behaviors in children and adolescents with autism spectrum disorders: a meta-analysis
Source: Front Psychiatry. 2026 May 19;17:1805431. doi: 10.3389/fpsyt.2026.1805431 (PMC13226569; doi:10.3389/fpsyt.2026.1805431)
Supplement: Supplementary file 2 [file DataSheet2.pdf]

Author(s): Yi Jingyao, Song Dongpo, Liu Wanxu, Huang Siyi, Li Xiaofen
Question: The impact of dance activities on social skills in children and adolescents with autism spectrum disorders: A systematic review and meta-analysis
Setting: Children and adolescents with autism spectrum disorders (ASD)
Bibliography: GUYATT, G. H., OXMAN, A. D., SCHUENEMANN, H. J., TUGWELL, P. & KNOTTNERUS, A. (2011), "GRADE guidelines : A new series of articles in the Journal of Clinical Epidemiology", JOURNAL OF CLINICAL EPIDEMIOLOGY, Vol. 64 No. 4, pp. 380-382. doi:10.1016/j.jclinepi.2010.09.011

Certainty assessment							№ of patients		Effect		Certainty	Importance
№ of studies	Study design	Risk of bias	Inconsistency	Indirectness	Imprecision	Other considerations	dance activities	[comparison]	Relative (95% CI)	Absolute (95% CI)		
**social skills**												
14	randomised trials	serious	serious	not serious	serious	publication bias strongly suspected strong association all plausible residual confounding would reduce the demonstrated effect dose response gradient	150/-	162/-	not estimable		⊕⊕⊕◯ Moderate	CRITICAL
**Communication abilities**												
10	randomised trials	serious	serious	not serious	serious	strong association all plausible residual confounding would reduce the demonstrated effect	97/-	103/-	not estimable		⊕⊕⊕◯ Moderate	CRITICAL
**Social Interaction**												
8	randomised trials	serious	serious	not serious	serious	strong association all plausible residual confounding would reduce the demonstrated effect	76/-	82/-	not estimable		⊕⊕⊕◯ Moderate	CRITICAL
**Repetitive behavior**												
7	randomised trials	serious	serious	serious	serious	strong association all plausible residual confounding would reduce the demonstrated effect	62/-	62/-	not estimable		⊕⊕◯◯ Low	CRITICAL
**Perceptual abilities**												
6	randomised trials	serious	serious	not serious	very serious	strong association all plausible residual confounding would reduce the demonstrated effect	61/-	61/-	not estimable		⊕⊕◯◯ Low	CRITICAL

CI: confidence interval
