# Supplementary material for: The impact of dance activities on social skills and related behaviors in children and adolescents with autism spectrum disorders: a meta-analysis
Source: Front Psychiatry. 2026 May 19;17:1805431. doi: 10.3389/fpsyt.2026.1805431 (PMC13226569; doi:10.3389/fpsyt.2026.1805431)
Supplement: Supplementary file 3 [file Supplementaryfile1.pdf]

Search strategy

PubMed 18

#1: ((((Autism Spectrum Disorder [MeSH Terms]) OR (Autism Spectrum Disorders)) OR (Autistic Spectrum Disorder)) OR (Autistic Spectrum Disorders)) OR (Disorder, Autistic Spectrum)

#2: ((((((((((((((((((((Dancing[MeSH Terms]) OR (dancing[Title/Abstract])) OR (Dance [Title/Abstract])) OR (Ballet[Title/Abstract])) OR (Jazz Dance[Title/Abstract])) OR (Dance, Jazz[Title/Abstract])) OR (Tap Dance[Title/Abstract])) OR (Dance, Tap[Title/Abstract])) OR (Modern Dance[Title/Abstract])) OR (Dance, Modern[Title/Abstract])) OR (Hip-Hop Dance [Title/Abstract])) OR (Dance, Hip-Hop[Title/Abstract])) OR (Hip Hop Dance[Title/Abstract])) OR (Line Dancing[Title/Abstract])) OR (Dancing, Line[Title/Abstract])) OR (Salsa Dancin g[Title/Abstract])) OR (Dancing, Salsa[Title/Abstract])) OR (Square Dance[Title/Abstract])) OR (Dance, Square[Title/Abstract])) OR (Dance Therapy[Title/Abstract])) OR (Dance move ment[Title/Abstract])) OR (Therapy, Dance[Title/Abstract])) OR (Dance Therapies[Title/Abst ract])) OR (Therapies, Dance[Title/Abstract])

#3: ((((((((((((((((((((((Child[MeSH Terms]) OR (Adolescent[MeSH Terms])) OR (You ng Adult[MeSH Terms])) OR (Minor[MeSH Terms])) OR (Adolescen[Title/Abstract])) OR (Teen[Title/Abstract])) OR (Teenager[Title/Abstract])) OR (Youth[Title/Abstract])) OR (Mino rs[Title/Abstract])) OR (Minor[Title/Abstract])) OR (Child[Title/Abstract])) OR (Child[Title/ Abstract])) OR (Kid[Title/Abstract])) OR (Kids[Title/Abstract])) OR (Girl[Title/Abstract])) O R (Boy[Title/Abstract])) OR (Under age[Title/Abstract])) OR (Underage[Title/Abstract])) OR (Young people[Title/Abstract])) OR (young person[Title/Abstract])) OR (Pubescen[Title/Abs tract])) OR (Young Adult[Title/Abstract])) OR (School age[Title/Abstract])) OR (Preschool [Title/Abstract])) OR (Student[Title/Abstract])

#4: #1 AND #2 AND #3

Cochrane 20

#1: Autism Spectrum Disorder

#2: (Disorder, Autistic Spectrum):ti,ab,kw OR (Autistic Spectrum Disorders):ti,ab,kw OR (Autistic Spectrum Disorder):ti,ab,kw OR (Autism Spectrum Disorders)

#3: Dancing

#4: (Dance, Square):ti,ab,kw OR (Square Dance):ti,ab,kw OR (Modern Dance):ti,ab,kw OR (Dance, Modern):ti,ab,kw OR (Dancing, Salsa):ti,ab,kw OR (Salsa Dancing):ti,ab,kw OR (Jazz Dance):ti,ab,kw OR (Dance, Jazz):ti,ab,kw OR (Ballet):ti,ab,kw OR (Line Dancing):ti,ab,kw OR (Dancing, Line):ti,ab,kw OR (Dance):ti,ab,kw OR (Hip Hop Dance):ti,ab,kw OR (Dance, Hip-Hop):ti,ab,kw OR (Hip-Hop Dance):ti,ab,kw OR (Tap Dance):ti,ab,kw OR (Dance, Tap)

#5: Child OR Adolescent OR Young Adult OR Minor

#6: (Children):ab,ti,kw OR (Children):ab,ti,kw OR (Child):ab,ti,kw OR (Adolescent):ab,ti,kw OR (Young Adult):ab,ti,kw OR (Minor):ab,ti,kw OR (Adolescen):ab,ti,kw OR (Teen):ab,ti,kw OR (Teenager):ab,ti,kw OR (Youth):ab,ti,kw OR (Minors):ab,ti,kw OR (Minor):ab,ti,kw OR (Child):ab,ti,kw OR (Kid):ab,ti,kw OR (Girl):ab,ti,kw OR (Boy):ab,ti,kw OR (Under age):ab,ti,kw OR (Underage):ab,ti,kw OR (Young people):ab,ti,kw OR (young person):ab,ti,kw OR (Prepubescen):ab,ti,kw OR (Pubescen):ab,ti,kw OR (Young Adult):ab,ti,kw OR (School age):ab,ti,kw OR (Preschool):ab,ti,kw OR (Student):ab,ti,kw

#7: #1 OR #2

#8: #3 OR #4

#9: #5 OR #6

#10: #7 AND #8 AND #9

Embase 78

#1: Autism

#2: 'Autism':ab,kw,ti OR 'autism spectrum disorder':ab,kw,ti OR 'autism, early infantile':ab,kw,ti OR 'autism, infantile':ab,kw,ti OR 'autistic child':ab,kw,ti OR 'autistic children':ab,kw,ti OR 'autistic disorder':ab,kw,ti OR 'autistic spectrum disorder':ab,kw,ti OR 'child development disorders, pervasive':ab,kw,ti OR 'childhood autism':ab,kw,ti OR 'classical autism':ab,kw,ti OR 'early infantile autism':ab,kw,ti OR 'infantile autism':ab,kw,ti OR 'infantile autism, early':ab,kw,ti OR 'Kanner syndrome':ab,kw,ti OR 'PDD (pervasive developmental disorder)':ab,kw,ti OR 'pervasive child development disorders':ab,kw,ti OR 'pervasive developmental disorder':ab,kw,ti OR 'pervasive developmental disorders':ab,kw,ti OR 'typical autism':ab,kw,ti OR 'autism':ab,kw,ti OR Asperger syndrome:ab,kw,ti OR 'Asperger disorder':ab,kw,ti OR 'Asperger`s disorder':ab,kw,ti OR 'Asperger`s syndrome':ab,kw,ti OR 'Aspergers disorder':ab,kw,ti OR 'Aspergers syndrome':ab,kw,ti OR 'high functioning autism':ab,kw,ti OR 'Asperger syndrome':ab,kw,ti

#3: Dancing

#4: 'dance':ab,kw,ti OR 'dancer':ab,kw,ti OR 'dancing':ab,kw,ti OR 'Dance therapy':ab,kw,ti OR 'dance movement psychotherapy':ab,kw,ti OR 'dance movement therapy':ab,kw,ti OR 'dance psychotherapy':ab,kw,ti OR 'dance therapy':ab,kw,ti

#5：'child'/exp OR 'child'

#6：'children':ab,kw,ti OR 'adolescent':ab,kw,ti OR 'adolescen':ab,kw,ti OR 'teen':ab,kw,ti OR 'teenager':ab,kw,ti OR 'youth':ab,kw,ti OR 'minors':ab,kw,ti OR 'minor':ab,kw,ti OR 'child':ab,kw,ti OR 'kid':ab,kw,ti OR 'girl':ab,kw,ti OR 'boy':ab,kw,ti OR 'under age':ab,kw,ti OR 'underage':ab,kw,ti OR 'young people':ab,kw,ti OR 'young person':ab,kw,ti OR 'prepubescen':ab,kw,ti OR 'pubescen':ab,kw,ti OR 'young adult':ab,kw,ti OR 'school age':ab,kw,ti OR 'preschool':ab,kw,ti OR 'student':ab,kw,ti

#7: #1 OR #2

#8: #3 OR #4

#9: #5 OR #6

#10: #7 AND #8 AND #9

WOS: 148

(TS=(Autism Spectrum Disorder) OR AB=(Autism Spectrum Disorders OR Autistic Spectrum Disorder OR Autistic Spectrum Disorders OR Disorder, Autistic Spectrum)) AND (TS=(Dancing) OR AB=(dancing OR Dance OR Ballet OR Jazz Dance OR Dance, Jazz OR Tap Dance OR Dance, Tap OR Modern Dance OR Dance, Modern OR Hip-Hop Dance OR Dance, Hip-Hop OR Hip Hop Dance OR Line Dancing OR Dancing, Line OR Salsa Dancing OR Dancing, Salsa OR Square Dance OR Dance, Square OR Dance Therapy OR Dance movement OR Therapy, Dance OR Dance Therapies OR Therapies, Dance)) AND (TS=(Child) OR TS=(Adolescent) OR TS=(Young Adult) OR TS=(Minor) OR TS=(Adolescen) OR AB=(Teen OR Teenager OR Youth OR Minors OR Minor OR Children OR Kid OR Kids OR Girl OR Boy OR Under age OR Underage OR Young people OR young person OR Prepubescen OR Pubescen OR Young Adult OR School age OR Preschool OR Student))

Ebsco 31

AB Autism Spectrum Disorder OR AB Autism Spectrum Disorders OR AB Autistic Spectrum Disorder OR AB Autistic Spectrum Disorders OR AB Disorder, Autistic Spectrum

AB Dancing OR AB dancing OR AB Dance OR AB Ballet OR AB Jazz Dance OR AB Dance, Jazz OR AB Tap Dance OR AB Dance, Tap OR AB Modern Dance OR A B Dance, Modern OR AB Hip-Hop Dance OR AB Dance, Hip-Hop OR AB Hip Hop Da nce OR AB Line Dancing OR AB Dancing, Line OR AB Salsa Dancing OR AB Dancin g, Salsa OR AB Square Dance OR AB Dance, Square OR AB Dance Therapy OR AB D ance movement OR AB Therapy, Dance OR AB Dance Therapies OR AB Therapies, Dan ce

AB Child OR AB Adolescent OR AB Young Adult OR AB Minor OR Adolescen O R AB Teen OR AB Teenager OR AB Youth OR AB Minors OR AB MinOR OR AB C hild OR AB Child OR AB Kid OR AB Kids OR AB Girl OR AB Boy OR AB Under

age OR AB Underage OR AB Young people OR AB young person OR AB Pubescen OR AB Young Adult OR AB School age OR AB Preschool OR AB Student
